# Potential microRNA-related targets in clearance pathways of amyloid-β: novel therapeutic approach for the treatment of Alzheimer’s disease

**DOI:** 10.1186/s13578-019-0354-3

**Published:** 2019-11-12

**Authors:** Soheil Madadi, Heidi Schwarzenbach, Massoud Saidijam, Reza Mahjub, Meysam Soleimani

**Affiliations:** 1 Department of Pharmaceutical Biotechnology, School of Pharmacy, Hamadan University of Medical Sciences, Hamadan, Iran; 2 Department of Tumor Biology, University Medical Center Hamburg-Eppendorf, 20246 Hamburg, Germany; 3 Department of Genetics and Molecular Medicine, Hamadan University of Medical Sciences, Hamadan, Iran; 4 Department of Pharmaceutics, School of Pharmacy, Hamadan University of Medical Sciences, Hamadan, Iran

**Keywords:** Ubiquitin–proteasome system, Autophagy, Aβ-degrading proteases, BBB transporters, Phagocytosis, Heat shock proteins, microRNAs

## Abstract

Imbalance between amyloid-beta (Aβ) peptide synthesis and clearance results in Aβ deregulation. Failure to clear these peptides appears to cause the development of Alzheimer’s disease (AD). In recent years, microRNAs have become established key regulators of biological processes that relate among others to the development and progression of neurodegenerative diseases, such as AD. This review article gives an overview on microRNAs that are involved in the Aβ cascade and discusses their inhibitory impact on their target mRNAs whose products participate in Aβ clearance. Understanding of the mechanism of microRNA in the associated signal pathways could identify novel therapeutic targets for the treatment of AD.

## Introduction

Alzheimer’s disease (AD)—the most common form of dementia—is a devastating diagnosis that accounts for 93,541 deaths in the United States in 2014 [1]. Clinical manifestation of AD is often a loss of memory and cognitive skills. AD comprises two types: early-onset AD (EOAD), the familial type of AD which is inherited in an autosomal dominant pattern, and sporadic late-onset AD (LOAD), the most prevalent form of AD which develops at a later age [2]. The main pathological characteristics in the brains of AD patients are extracellular senile plaques composed of Aβ peptides [3] and intracellular neurofibrillary tangles (NFTs) formed by the accumulation of hyperphosphorylated tau [4].

Aβ is cleaved from the amyloid precursor protein (APP) by β-secretase (BACE1) and γ-secretase in the amyloidogenic pathway [5], while in the non-pathological stage, APP is cleaved to non-toxic proteins by α-secretase [6]. Aβ has two major forms: Aβ40 and Aβ42, which are 40 and 42 amino acid-long fragments, respectively. Since Aβ42 is more hydrophobic than Aβ40, it is more prone to aggregate and scaffold for oligomeric and fibrillar forms [7]. The microtubule-associated protein tau regulates the assembly of microtubules and maintains its structural stability. Thus, it plays an important role in microtubule dynamics. In AD, however, tau becomes abnormally hyperphosphorylated leading to its dissociation from microtubules. Then, the unbound tau molecules aggregate as insoluble filaments, which accumulate and form neurofibrillary tangles (NFT) [8]. The accumulation of Aβ and NFTs in brain can trigger a cascade of events that may lead to AD.

**Fig. 1:**
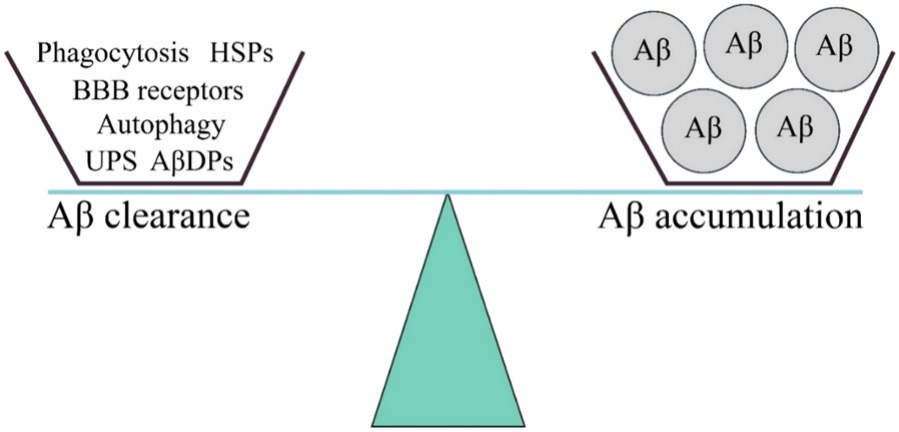
Balanced Aβ clearance pathways. *UPS* ubiquitin–proteasome system, *AβDPs* Aβ degrading proteases, *BBB* blood brain barrier, *HSP* heat shock proteins

According to the Aβ hypothesis, Aβ accumulation arises from a failure of clearance rather than over-production [9]. Indeed, Bateman et al. [10] demonstrated that the clearance rate of Aβ is impaired by approximately 30% in the cerebrospinal fluid of patients with LOAD. Mawuenyega et al. [11] found that the clearance rate of Aβ40 and Aβ42 is reduced by 25% and 30%, respectively in AD patients. The study by Cirrito et al. [12] showed the effect of age on the clearance rate of Aβ and found that the half-life of Aβ doubled within the interstitial fluid of older animal models of AD. These studies definitely established that defects in Aβ clearance have a fundamental role in AD pathology. Mechanisms that are involved in Aβ clearance include the ubiquitin–proteasome system (UPS), autophagic processes, proteolytic enzymes, transportation across the blood brain barrier (BBB), cellular uptake and heat shock protein (HSP)-mediated clearance, as illustrated in Fig. 1. The relative contributions of each of these procedures resulting in the overall clearance of Aβ are unknown.

MicroRNAs (miRNAs) have emerged as essential post-transcriptional regulators of gene expression. These small, non-coding RNAs regulate mRNA stability and transcription by binding to the 3′-UTR region of their targets [13]. The dysregulation of miRNAs leads to an altered protein expression which in turn results in a pathogenic signaling network connected with the imbalance between Aβ peptide synthesis and clearance causing AD. The involvement of miRNAs in these pathways may provide information about the molecular mechanism of AD. To survey and overcome the imbalance between synthesis and clearing, the research field on miRNAs may be promising, and is eligible for establishing a continuous monitoring of disease progression and therapeutic interventions, not only for AD but also for other diseases.

To date, miRNAs described above document their usefulness as diagnostic and predictive markers for AD. For the assessment of miRNAs, real-time PCR, microarrays or even sequencing could be applied in tissues and body fluids, such as plasma or serum. The development of miRNA-based therapies anticipates restoring normal miRNA expression levels. In clinical settings, the levels of down-regulated tumor suppressor miRNAs could be normalized by their re-expression using synthetic or viral vectors encoded for miRNA or synthetic double strand RNA molecules (mimics), whereas the up-regulated oncogenic miRNAs could be silenced by antisense-mediated inhibition, miRNA sponges and anti-miRNA peptides. As delivery vehicles of miRNAs could serve polymer-based, lipid or viral vesicles or MSCs [14]. However, to reach their destination, miRNAs (mimics or antisense) have to cross the blood–brain barrier. To overcome this limitation, strategies, such as the use of conjugated nanoparticle or intracerebroventricular infusion have been shown to improve the transport through the blood–brain barrier [15]. Further challenges for an efficient miRNA-based gene therapy are the potential degradation of miRNAs by cellular nucleases and poor cellular uptake. In particular, miRNAs elicit unspecific effects, toxicity and/or unfavorable immune response, since they only partially bind to their target mRNA. In addition, they participate in several signaling pathways and consequently, have different regulatory functions which require further research. For example, with respect to the treatment of cancer, in September 2016, the sponsoring company (Mirna Therapeutic, Inc.) stopped the enrollment and dosing of miR-34 (MRX34) in a clinical study after numerous immune-related severe adverse effects in patients dosed with MRX34 [16]. Therefore, to realize their therapeutic application, it is essential to intensely investigate the biology and functions of miRNAs. As described above, numerous efforts have already made to identify miRNAs for introducing them into the clinical practice of AD. Most notably in animal models, these miRNAs appeared to be well tolerated with promising outcomes. For example, the intracerebroventricular infusion of anti-miR-33 inhibited the brain-specifically expressed miR-33 and in turn decreased Aβ levels in the cortex of mice [17].

On the other hand, a disruption of miRNA biogenesis is to avoid since it is assumed to cause neurodegeneration. For example, the onset of a neurodegenerative disease may happen by the loss of Dicer, an enzyme which cleaves pre-miRNA into a double-stranded miRNA duplex [18]. Such investigations show that miRNAs play an important role in long-term brain integrity and highlight their clinical relevance in AD. As up to 80% of all human genes are regulated by miRNAs [19] and their potential utility as AD biomarkers have been reported, we introduce potential miRNA-regulated targets in Aβ clearance pathways that will provide insights into the role of miRNAs in AD pathology.

## Ubiquitin–proteasome system

The ubiquitin–proteasome system (UPS) is the main intracellular proteolytic pathway in eukaryotic cells. The pathway degrades more than 70–80% of intracellular proteins, including damaged and misfolded proteins [20]. At first, in the tagging reaction of the UPS-mediated protein degradation, a polyubiquitin chain is added to target proteins through three steps: (1) in an ATP-dependent process, an ubiquitin-activating enzyme (E1) activates an ubiquitin (Ub) monomer, a 76-amino acid peptide; (2) the activated Ub binds to an ubiquitin-conjugating enzyme (E2); and (3) ubiquitin ligase (E3) then transfers Ub to the target protein. In some cases, an additional ubiquitination enzyme, the chain elongation factor E4, is required to extend a polyubiquitin chain. Finally, the polyubiquitinated proteins are recognized and degraded in the 26S proteasome, a system that is composed of a 20S catalytic core and two 19S regulatory subunits [21].

After the detection of Ub in senile plaques in 1987 [22] and the observation that Aβ can bind to proteasomes [23], it was suggested that UPS is involved in the clearance of Aβ. Later studies substantiated this hypothesis. Lopez et al. [24] demonstrated that inhibition of the proteolytic activity of the 26S proteasome in neurons and astrocytes led to a reduction in Aβ degradation. Chadwick et al. [25] showed that a mutant form of Ub capped by polyubiquitin chains inhibited 26S proteasome and interfered with Aβ clearance. Furthermore, proteolytic activities of the 26S proteasome can also be inhibited by Aβ [26].

### MiRNAs and their targets in UPS

Usually, in neocortex and hippocampal regions of AD brain tissues, the E2 family member UBE2A is down-regulated. In this regard, Zhao et al. [27] showed that the over-expression of miR-7 led to UBE2A down-regulation in the brain tissues of AD patients. In addition, the E2 isoforms UBE2B, UBE2D3 and UBCH10 that were down-regulated by miR-455-5p [28], miR-21-5p [29] and miR-631 [30] respectively, were identified as AD-related genes in a study conducted by Libro et al. [31]. Finally, the expression of UBC9 (UBE2I) was inversely correlated with miR-30a and miR-214 expression [32, 33] (Table 1).

**Table 1:** MiRNAs and their downregulated mRNA targets in UPS

MiRNAs	Family	Gene	References
miR-199a-5p	Ubiquitin-conjugating (E2) enzymes	UBE2G1	[45]
miR-101		UBE2N	[46]
miR-182, miR-145, miR-19a/b	Ubiquitin E3 ligases	CUL5	[47–49]
miR-195		CBX4	[50]
miR-221		HECTD2	[51]
miR-153		HECTD3	[52]
miR-542-5p		HUWE1	[53]
miR-106b, miR-411		ITCH	[54, 55]
miR-93		NEDD4L	[56]
miR-137		PIAS2	[57]
miR-199a-5p, miR-301a-3p, miR-9718, miR-21, miR-18a		PIAS3	[58–62]
miR-194		RBX1	[63]
miR-503, miR-542-5p, miR-497, miR-15b		SMURF1	[64–67]
miR-486, miR-424, miR-322, miR-503, miR-15a/b, miR-16, miR-128		SMURF2	[68–71]
miR-542-3p		UBE3C	[72]
miR-584-5p, miR-21		WWP1	[73, 74]
miR-214		RNF8	[75]
miR-19b		MYLIP	[76]
miR-214		RFWD2	[77]
miR-31	Deubiquitinating enzymes	BAP1	[78]
miR-17		USP2	[79]
miR-148a		USP4	[80]
miR-205		USP7	[81]
miR-135b		USP13	[82]
miR-320a		USP14	[83]
miR-34b		USP22	[84]
miR-200c		USP25	[85]
miR-363-3p		USP28	[86]
miR-204-5p		USP47	[87]
miR-25		Ataxin-3	[88]
miR-125b-5p		A20	[89]
miR-24		CSN5	[90]

There are several hundred E3 ligases in mammals, and this class shows the greatest diversity among the enzymes. E3 ligases are divided into two classes: E3 ligases with homology to the E6-AP carboxyl terminus (HECT), and the new RING ligases [34]. Singh et al. showed that the decreased levels of E3 ligase UBE3A caused by miR-375 over-expression [35], could influence the progression of AD [36]. Christie et al. showed that the levels of E3 ligase XIAP which were down-regulated by miR-497 and miR-7 [37, 38], were higher in AD patients than control cases [39]. Similarly, miR-24 over-expression decreased XIAP expression [40] (Table 1).

**Fig. 2:**
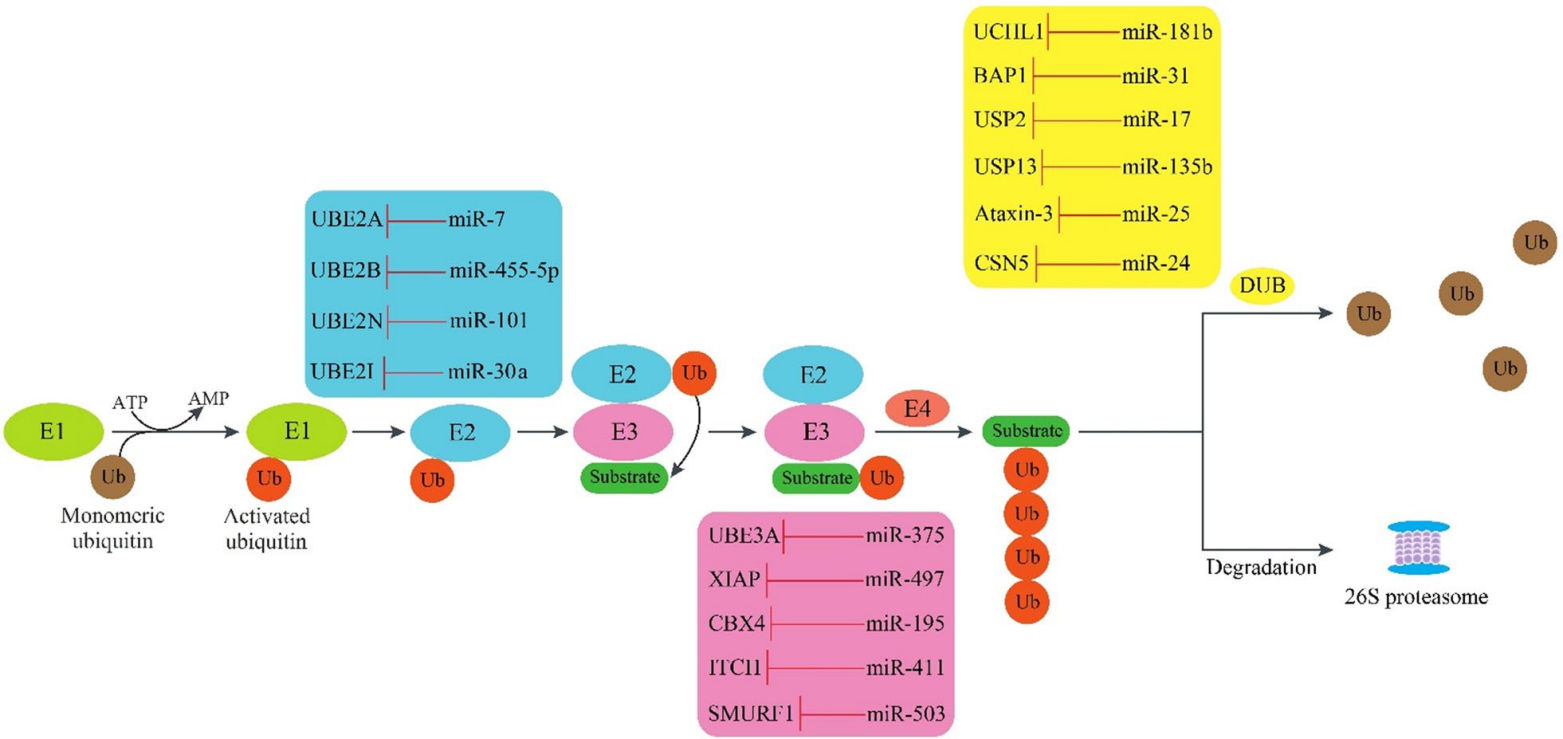
The inhibitory effect of miRNAs on their target molecules in the UPS pathway. Ubiquitin is transferred to the E2 enzyme after activation by the E1 enzyme, and is then transferred to the substrate by E3 enzyme. E4 enzyme is required for the formation of the polyubiquitin chain. After the recognition process, substrates are degraded by the 26S proteasome or their polyubiquitin monomers are removed by DUB. *Ub* ubiquitin, *E1* ubiquitin-activating enzyme, *E2* ubiquitin-conjugating enzyme, *E3* ubiquitin ligase, *DUB* deubiquitinating enzyme, *UBE2A* ubiquitin conjugating enzyme E2 A, *UBE2B* ubiquitin conjugating enzyme E2 B, *UBE2C* ubiquitin conjugating enzyme E2 C, *UBE2I* ubiquitin conjugating enzyme E2 I, *UBE3A* E3 ubiquitin-protein ligase A, *XIAP* E3 ubiquitin-protein ligase XIAP, *CBX4* E3 SUMO-protein ligase CBX4, *ITCH* E3 ubiquitin-protein ligase Itchy, *SMURF1* E3 ubiquitin-protein ligase SMURF1, *UCHL1* ubiquitin carboxyl-terminal hydrolase isozyme L1, *BAP1* ubiquitin carboxyl-terminal hydrolase BAP1, *USP2* ubiquitin specific peptidase 2, *USP13* ubiquitin specific peptidase 13; *CSN5* COP9 signalosome complex subunit 5

There are ~ 95 deubiquitinating enzymes (DUBs) in the human genome. DUBs are classified into five classes including: ubiquitin C-terminal hydrolase (UCH), ubiquitin-specific protease (USP), Machado-Joseph disease protease (MJD), otubain protease (OTU) and JAB1/MPN/Mov34 metalloenzyme (JAMM) [41]. Ubiquitin C-terminal hydrolase L1 (UCHL1) appears to be the only DUB playing a role in AD. It constitutes 1–5% of total neuronal protein, and stabilizes monoubiquitin by binding to it [42]. MiR-922 and miR-181b decreased UCHL1 expression in kidney and neuroblastoma cells, respectively [43, 44] (Table 1; Fig. 2).

## Autophagy

Autophagy is a highly conserved catabolic process which has a key role in maintaining cell hemostasis through recycling nutrients and degrading aggregated proteins or damaged organelles [91]. Autophagy has distinct stages: formation of an isolation membrane (phagophore) and initiation of autophagy, vesicle nucleation, elongation and expansion of the autophagosome membrane, sequestration of aggregated proteins and cytoplasmic organelles into an autophagosome, and finally fusion of autophagosomes with endosomes or lysosomes for content degradation.

The first step in the autophagy process is the fusion of vesicles that originate from different membrane sources, such as the plasma membrane, endoplasmic reticulum (ER), Golgi apparatus and mitochondria [92]. Integration of these vesicles leads to the formation of an isolation membrane, called the phagophore. Autophagy initiation begins with the activation of a complex comprised of ULK1, ULK2, ATG13, ATG101 and the family interacting protein of 200 kD (FIP200) [93]. The mechanistic target of the rapamycin complex 1 (mTORC1) which is comprised of mTOR, RAPTOR, mLST8, and DEPTOR inhibits autophagy by phosphorylating ULK1 and ATG13 [94], while the adenosine monophosphate activated protein kinase (AMPK) activates autophagy by phosphorylating ULK1 at other sites [94].

The ULK1 complex controls vesicle nucleation through the class III phosphatidylinositol 3-kinase (PI3 K) complex. This complex is comprised of vacuolar protein sorting 34 (VPS34), VPS15, ATG14, and ultraviolet irradiation resistance-associated gene (UVRAG), all of which are scaffolded by Beclin 1 [95]. There are two ubiquitin-like conjugation steps that are involved in autophagosome elongation: (1) formation of a complex between ATG5, ATG12 and ATG16L1 that requires the catalytic activities of ATG7 (E1-like enzyme) and ATG10 (E2-like enzyme), (2) processing of microtubule-associated protein 1 light chain 3 (LC3). Initially, LC3 is cleaved by ATG4B, to form LC3-I which is then conjugated to phosphatidylethanolamine (PE) by ATG7 (E1-like enzyme) and ATG3 (E2-like enzyme), to form LC3-II [96]. After the formation of autophagosomes, the ATG5-ATG12-ATG16L1 complex separates from the outer membrane, while LC3-II remains attached with the completed autophagosomes, to facilitate their identification. Finally, double-membraned autophagosomes fuse with lysosomes for content degradation.

**Fig. 3:**
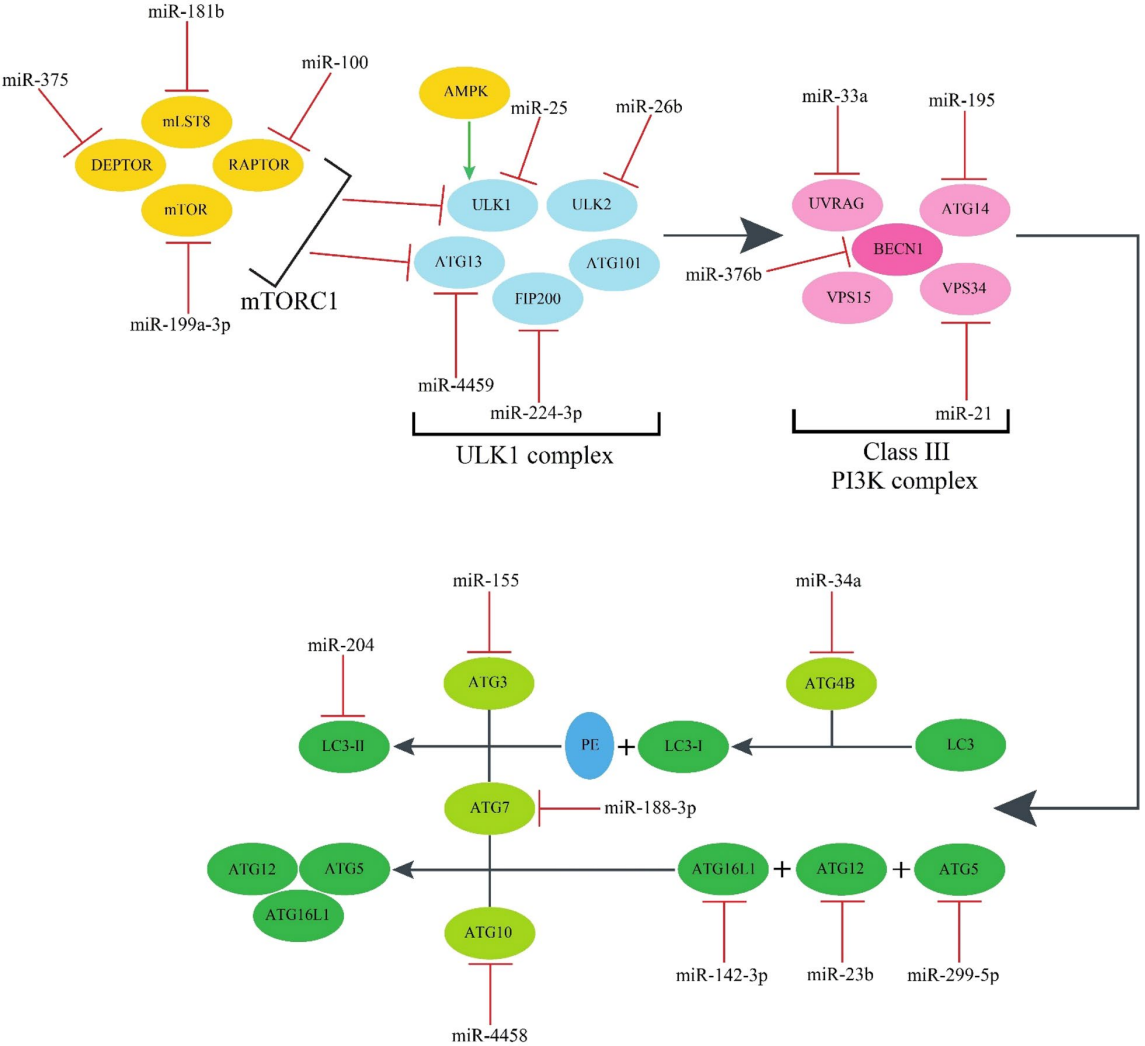
MiRNAs inhibit autophagy by down-regulating their target molecules. AMPK and mTORC1 are key modulators of autophagy and exert their effects by regulating ULK1 and ATG13. Activation of the ULK1 complex initiates autophagy and regulates vesicle nucleation through the class III phosphatidylinositol 3-kinase (PI3K) complex. The final step in the autophagosome formation requires the two ubiquitin-like conjugation systems. *AMPK* adenosine monophosphate activated protein kinase, *mTOR* mechanistic target of rapamycin, *RAPTOR* regulatory-associated protein of Mtor, *mLST8* MTOR associated protein, LST8 homolog, *DEPTOR* DEP domain containing MTOR interacting protein, *ULK1* unc-51 like autophagy activating kinase 1, *ULK2* unc-51 like autophagy activating kinase 2, *ATG* autophagy-related gene, *FIP200* family interacting protein of 200 kD, *UVRAG* UV radiation resistance-associated gene protein, *LC3* microtubule-associated protein 1 light chain 3, *PE* phosphatidylethanolamine

Growing evidence indicates that autophagy plays a role in AD pathology. For example it has been reported that autophagic vacuoles are abundant in AD brains [97] and that their clearance is impaired in AD [98]. Furthermore, restoring autophagy reduced Aβ accumulation in a TgCRND8 mouse model of AD and ameliorated memory deficits [99]. In their study, Wu et al. [100] validated miRNA-binding sequences for miR-20a and miR-106b in the 3′-UTR region of ULK1 and found that these two miRNAs negatively regulated autophagy through suppressing ULK1 expression in mouse myoblast cell lines. Korkmaz et al. [101] found that miR-376b attenuated the luciferase activity of the BECN1 3′-UTR, and thus, decreased mRNA levels of BECN1 in human breast and hepatocellular carcinoma cell lines leading to autophagy inhibition. A number of miRNAs that regulate the autophagy cascade are summarized in Table 2, Fig. 3.

**Table 2:** MiRNAs and their downregulated mRNA targets in the autophagy cascade

MiRNA	Function	Gene	References
miR-144, miR-99b-5p, miR-199a-3p	Autophagy initiation	mTOR	[102–104]
miR-100		RAPTOR	[105]
miR-181b		mLST8	[106]
miR-375		DEPTOR	[107]
miR-25		ULK1	[108]
miR-26b		ULK2	[109]
miR-4459		ATG13	[110]
miR-224-3p		FIP200	[111]
miR-17-5p, miR-30a	Vesicle nucleation	BECN1	[112, 113]
miR-195, miR-152		ATG14	[114, 115]
miR-33a, miR-183		UVRAG	[116, 117]
miR-21		VPS34	[118]
miR-299-5p, miR-181a	Autophagosome elongation	ATG5	[119, 120]
miR-23b, miR-200b		ATG12	[121, 122]
miR-142-3p, miR-410		ATG16L1	[123, 124]
miR-188-3p, miR-17		ATG7	[125, 126]
miR-4458, miR-4667-5p, miR-4668-5p		ATG10	[127]
miR-34a		ATG4B	[128]
miR-155		ATG3	[129]
miR-204, miR-497		LC3-II	[130, 131]

## Degrading enzymes

Aβ is degraded by various types of proteases collectively known as Aβ-degrading proteases (AβDPs), e.g., by neprilysin, myelin basic protein, matrix metallopeptidase, angiotensin converting enzyme and cathepsins.

### Neprilysin

Neprilysin (NEP) is a zinc-dependent membrane metalloendopeptidase (MME) belonging to the M13 family of metallopeptidases. After the introduction of Neprilysin as one of the major AβDPs [132], Iwata et al. [133] showed that in Neprilysin knockout-mice the vulnerability of the hippocampus was caused by Aβ accumulation. In this regard, neprilysin was shown to degrade both monomeric and oligomeric forms of Aβ [134]. Moreover, a meta-analysis documented that mRNA and protein levels of Neprilysin, as well as the enzymatic activity of neprilysin are decreased in AD patients [135].

### Myelin basic protein

Myelin basic protein (MBP), an 18.5 kD protein is the main protein component of myelin, and participates in the formation and maintenance of the myelin sheath. MBP has serine protease activity and degrades Aβ40 and Aβ42 peptides [136]. Hoos et al. [137] found that MBP inhibited fibrillar assembly of Aβ, and Liao et al. [138] demonstrated that this was mediated by the N-terminal domain of MBP. Furthermore, Wang et al. [139] showed that miR-212 reduced the expression of MBP, and thus, promoted the assembly.

### Matrix metallopeptidase

Matrix metalloproteinases (MMPs) that belong to the metzincin family have at least two domains: the pro-domain which is ~ 80 amino acids long and the catalytic-domain which contains a zinc ion in the active site. They degrade both soluble and fibrillar Aβ peptides [140]. Zhang et al. [141] reported that miR-9 directly targeted the MMP-14 3′-UTR and decreased transcriptional and consequently, protein levels of MMP-14 in neuroblastoma cells reducing adhesion, migration, invasion and angiogenesis of these cells. Multiple MMPs are implicated in Aβ degradation and their repression by miRNAs is shown in Table 3.

**Table 3:** MiRNAs and their downregulated mRNA targets in the degradation cascade

MiRNA	Family	Gene	References
miR-24, miR-181a-5p	Matrix metalloproteinase	MMP-14	[142, 143]
miR-132, miR-34a, miR-516b		MMP-9	[144–146]
miR-148a, miR-100		MMP-7	[147, 148]
miR-29b, miR-34a, miR-516b, miR-93		MMP-2	[145, 146, 149, 150]
miR-22, miR-485-5p, miR-492		EMMPRIN	[151–153]
miR-143/145	Angiotensin-converting enzyme	ACE	[154]

### Angiotensin converting enzyme

Angiotensin-converting enzyme (ACE) is a zinc-dependent dipeptidase that catalyzes the conversion of angiotensin I to angiotensin II. Hu et al. [155] found that ACE degraded Aβ40 by cleaving the peptide bond between Asp7 and Ser8 residues, and found that ACE prevented the accumulation of amyloid plaques by degrading Aβ in vivo. Following studies indicated that the N-terminal domain of ACE was responsible for Aβ degradation [156] and pharmacological inhibition of ACE enhanced the accumulation of Aβ in APP expressing cells [157]. Several miRNAs are implicated in inhibiting ACE expression, as listed in Table 3.

### Cathepsins

Cathepsin B, a major representative of cysteine proteases, acts as either an exopeptidase or an endopeptidase. It is present in lysosomes from all cell types, and participates in lysosomal turnover of proteins. Sun et al. [158] indicated that Cathepsin B was able to induce Aβ degradation in vivo. Moreover, lysosomal Cathepsin B is essential in microglial clearance of Aβ [159] and its up-regulation promotes Aβ42 degradation in AD monocytes [160]. By using homology modeling, Dhanavade et al. [161] found that Cathepsin B cleaved Aβ peptide from the carboxylic end of Glu11. Cathepsin D, an aspartyl protease is present in lysosomes from most mammalian cells, and engages in the degradation of intracellular and endocytosed proteins. It cleaves Aβ peptide at Phe19-Phe20 and Leu34-Met35 [162], and is down-regulated in monocytes of AD patients [163]. Overexpression of miR-128 down-regulated the expression of Cathepsin B and Cathepsin D. Consequently, miR-128 inhibition enhanced Aβ42 degradation in monocytes from AD patients [164].

## Blood–brain barrier clearance of Aβ

**Fig. 4:**
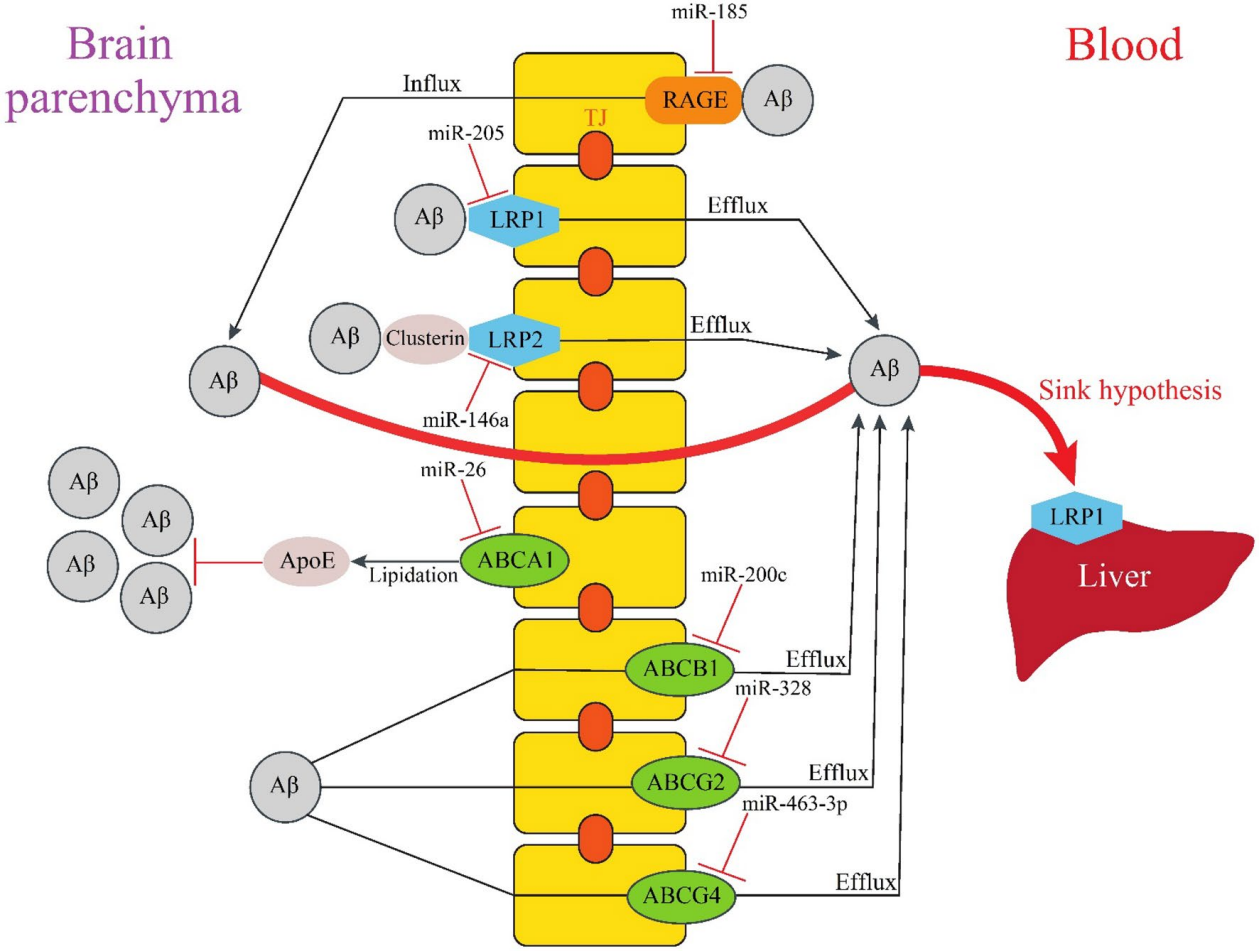
MiRNA-dependent regulation of Aβ clearance through the BBB. Aβ efflux and influx through the BBB. Aβ entrance to the brain is mediated by RAGE, but several transporters are expressed on both the luminal and abluminal sides of the BBB can eliminate Aβ from the brain. LRP1 is one of the major receptors for Aβ efflux through the BBB. Due to the sink hypothesis, it is also implicated in Aβ removal. Presence of Clusterin is essential for Aβ clearance by LRP2 receptor. ABCA1 indirectly facilitates Aβ clearance by lipidating ApoE. The drug pumps ABCB1 and ABCG2 are also involved in Aβ transport across the BBB. *RAGE* receptor for advanced glycation end products, *LRP1* LDL receptor related protein 1, *LRP2* LDL receptor related protein 2, *ABCA1* ATP binding cassette subfamily A member 1, *ABCB1* multidrug resistance protein, P-glycoprotein, *ABCG2* breast cancer resistance protein, *ABCG4* ATP binding cassette subfamily G member 4

The blood–brain barrier (BBB) is a physical barrier that separates peripheral circulation from the central nervous system (CNS). The BBB, which is formed by endothelial cells connected by tight junctions, plays a significant role in controlling brain homeostasis by eliminating toxic metabolites from the brain into the blood, such as Aβ aggregates. It has two sides, the luminal side facing the blood circulation, and the abluminal side facing the brain parenchyma. Transporters and receptors which are expressed on the two sides are involved in the transportation and clearance of Aβ. Aβ efflux and influx through the BBB are regulated by several miRNAs, some of which are illustrated in Fig. 4 and listed in Table 4.

**Table 4:** MiRNAs and their downregulated mRNA targets in the blood–brain barrier clearance of Aβ

MiRNA	Family	Gene	References
miR-185, miR-328-5p	Receptor for advanced glycation end products	RAGE	[220, 221]
miR-205	Low-density lipoprotein receptors	LRP1	[222]
miR-148b		LRP2	[223]
miR-26, miR-19b, miR-302a	ATP-binding cassette transporters	ABCA1	[224–226]
miR-200c, miR-873, miR-491-3p, miR-223		ABCB1	[227–230]
miR-328, miR-302, miR-3163, miR-181a		ABCG2	[231–234]
miR-185-5p, miR-463-3p		ABCG4	[235, 236]
miR-29b, miR-130b	Glymphatic clearance	AQP4	[237, 238]

### Receptor-mediated Aβ influx

#### Receptor for advanced glycation end products

The receptor for advanced glycation end products (RAGE) belongs to the immunoglobulin family, and is expressed on the luminal surface of brain vessels. RAGE is a multi-ligand receptor that binds a range of ligands, including Aβ [165]. By using an in vitro BBB model, Mackic et al. [166] showed that RAGE is involved in the internalization of soluble monomeric forms of Aβ40. Candela et al. [167] reported that RAGE inhibitors mediated a significant decrease in Aβ40 and Aβ42 transport through the brain endothelium. Similarly, Takuma et al. [168] found that a genetic deletion of RAGE suppressed Aβ uptake in neurons. Mice studies confirmed these findings and showed the influx of circulating Aβ into the brain as a receptor-mediated transport depending on RAGE [169]. Furthermore, the inhibition of the RAGE/Aβ interaction repressed Aβ accumulation in the brain of PD-hAPP mice [170]. Also in line with these data, Ma et al. showed that RAGE up-regulation contributed to the accumulation of Aβ and cognitive impairment in rats [171]. Finally, Choi et al. [172] reported the elevated RAGE expression in a mouse model of AD.

### Receptor-mediated Aβ efflux

#### Low-density lipoprotein receptor (LDLR) family

The LDLR family are cell surface receptors and includes LDLR, VLDLR, LRP1, LRP1B, LRP2 (megalin), LRP3, LRP4, LRP5, LRP6 and LRP8. The main function of this receptor family is receptor-mediated endocytosis. In APP/PS1/LDLR transgenic mice, LDLR over-expression was reported to promote Aβ clearance [173].

Initial studies identified LRP1 as an abluminal receptor that mediated Aβ transport across the BBB [174], and subsequent studies proved a role for LRP1 in brain-to-blood Aβ clearance [175]. In a mouse model of AD, LRP1 deletion resulted in decreased Aβ levels in plasma and enhanced soluble Aβ in brain endothelial cells [176]. Moreover, LRP1 oligodeoxynucleotide antisense impaired recognition memory in mice by reducing BBB clearance of Aβ [177]. Several studies proved that ApoE had suppressive effects on LRP1-mediated BBB clearance of Aβ as preincubation with ApoE reduced Aβ40 clearance [178]. Moreover, ApoE suppressed soluble Aβ (sAβ) clearance by competing with sAβ for interaction with LRP1 [179]. Further studies showed an isoform-specific effect for ApoE since ApoE4-Aβ complexes were not cleared by the rapid LRP1 receptor, and their clearance was mediated by VLDLR which has a significant slower rate of endocytosis compared to LRP1. However, both LRP1 and VLDLR are involved in the clearance of ApoE2- and ApoE3-Aβ complexes [180]. Wang et al. [181] found that miR-1908 reduced mRNA levels of ApoE by targeting its 3′-UTR, and thereby inhibited ApoE-mediated Aβ clearance in astrocytoma and human macrophage cell lines.

Based on the sink hypothesis, it is assumed that expression of LRP1 in peripheral tissues affects Aβ clearance through the BBB. According to this hypothesis, an equilibrium exists between the levels of Aβ in the brain and peripheral tissues. Thus, Aβ elimination by peripheral tissues causes brain Aβ to move into the blood through the BBB in order to maintain this balance [175, 182]. By expressing LRP1, the liver is able to clear plasma Aβ [183], therefore, LRP1 suppression in the liver reduced the Aβ uptake as reported by Tamaki et al. [184]. Clearance of plasma Aβ by the liver is saturable and age-related [184]. Investigations showed that soluble LRP1 which is produced from the cleavage of LRP1 by β-secretase [185], is the main peripheral Aβ-binding protein and reduced the load of Aβ in mice brain by acting as a peripheral sink [186].

LRP2 (megalin) is expressed on the abluminal side of the BBB, and also involved in the BBB clearance of Aβ [187]. Aβ does not directly bind to LRP2, and needs ApoJ for the interaction with LRP2 [188]. Only, ApoJ-bounded Aβ can be cleared from the brain by this receptor [189]. Interestingly, a recent study indicated that Clusterin administration reduced Aβ accumulation in a mouse model of AD by increasing LRP2 levels [190]. Zhang et al. [191] identified LRP2 mRNA 3′-UTR as a direct target of miR-146a and indicated that LRP2 protein levels were significantly inhibited by miR-146a in human neuroblastoma cell line. MiR-146a also elevated the rate of apoptosis in human neuroblastoma cells exposed to Aβ, and thus, may contribute to AD progression.

#### ATP-binding cassette transporters (ABC transporters)

The ABC transporter, one of the most common transmembrane proteins exists in all living organisms and is divided into subfamilies A to G based on its sequence homology and functional similarity. ABC transporters use the energy generated by ATP hydrolysis to transport substrates across cell-membranes, playing an important role in many physiological processes. Recent evidence showed that ABC transporters are involved in Aβ clearance, especially ABCA1, ABCB1 (multidrug resistance protein, MDR1 or *P*-glycoprotein), ABCG2 (breast cancer resistance protein, BCRP), and ABCG4.

ABCA1 is a transmembrane protein that is expressed on the abluminal side of the BBB. It transports cholesterol and phospholipids to ApoE in order to form high-density lipoproteins (HDL). Analyses showed that ABCA1 indirectly facilitated Aβ clearance through ApoE lipidation in the brain as no significant differentiation was seen in Aβ elimination between ABCA1-deficient and wild-type mice [192]. Mouse studies indicated that ABCA1 deficiency reduced ApoE levels and its lipidation state in the brain which were accompanied by Aβ accumulation [193, 194] and co-deposition of poorly lipidated ApoE with Aβ [195]. Thus, ABCA1-mediated ApoE lipidation reduced Aβ accumulation [196]. Similarly, Corona et al. [197] revealed that ABCA1-mediated ApoE lipidation is essential in Aβ clearance. The role of ABCA1 and ApoE in Aβ clearance is not fully elucidated as Aβ clearance was reduced in APP/ABCA1^+/−^ mice expressing ApoE4 but not ApoE3 [198]. While ABCA1 expression was reduced in the brain of APP/PS1 mice [199], it was up-regulated in 3xTg-AD mice [200]. Further studies showed that ABCA1-mediated cholesterol efflux was reduced in the CSF of AD patients [201]. Nordestgaard et al. [202] found that a loss-of-function mutation in ABCA1 was associated with a higher risk of AD. In neuroblastoma and liver cells, miR-106b prevented Aβ clearance by suppressing ABCA1 expression [203], while inhibition of miR-33a increased lipidated ApoE levels, and reduced Aβ levels mediated by the re-expression of ABCA1 [17]. Liang et al. [204] found that miR-20a/b reduced mRNA and protein expression of ABCA1 in human and mouse macrophage-derived foam cells. MiR-20a/b over-expression decreased cholesterol efflux to ApoA-I, and thus, may interfere with Aβ clearance.

The ABCB1 transporter that is expressed on the luminal side of the BBB acts as an efflux pump of exogenous molecules, and is involved in Aβ clearance, as shown in ABCB1-knockout mice [205]. Other in vitro and in vivo studies also proved that *P*-glycoprotein had efflux activity since ABCB1 up-regulation enhanced the efflux of Aβ40 from cells [206] and led to a reduction in parenchymal Aβ40 and Aβ42 levels [207]. Moreover, previous studies showed that peripherally-injected Aβ accumulated in the brain of ABCB1-knockout mice [208], and ABCB1 deficiency increased Aβ burden in a mouse model of AD [209]. Consistent with these results, Aβ accumulation was inversely correlated with ABCB1 expression in AD patients [210]. Notably, Aβ42 down-regulated the expression of *P*-glycoprotein [211].

ABCG2 is also expressed at the luminal side of the BBB, and is also involved in Aβ efflux from brain to blood circulation [212] since Aβ levels were reported to be higher in the brain of ABCG2 knock-out mice than in the brain of wild type mice [208]. Shen et al. [213] also proved that ABCG2 had efflux activity, since ABCG2 deficiency led to Aβ accumulation in mice brain. Moreover, ABCG2 levels were age-dependently increased in a mice model of AD [200], and Xiong et al. [214] reported its up-regulation in AD brains.

The ABCG4 transporter participates in the cholesterol and desmosterol efflux. Do et al. [200] identified ABCG4 as a receptor that controls Aβ efflux through the BBB. Other in vivo studies proved its role in Aβ clearance by disclosing that ABCG4 contributes to Aβ40 elimination across the mouse BBB [212], and that Aβ efflux was decreased in ABCG4-knockout mice [215]. Finally, a mouse model showed that ABCG4 is expressed in the cerebral cortex and medulla regions of the brain [216], while a human study demonstrated that ABCG4 was up-regulated in the microglia-surrounded senile plaques in AD brains [217].

### Glymphatic clearance

Aquaporin-4 (AQP4), a water-channel protein is expressed in astrocytes, and plays a key role in Aβ clearance by regulating the glymphatic pathway. AQP4 is involved in the clearance of soluble Aβ from the brain [218]. Yang et al. [219] revealed that AQP4 was up-regulated in areas of senile plaques, predominantly at later stages of plaque formation. In AQP4 knockout mice, glymphatic clearance of Aβ was reduced compared with wild-type mice [218].

## Receptor-mediated Aβ phagocytosis

**Fig. 5:**
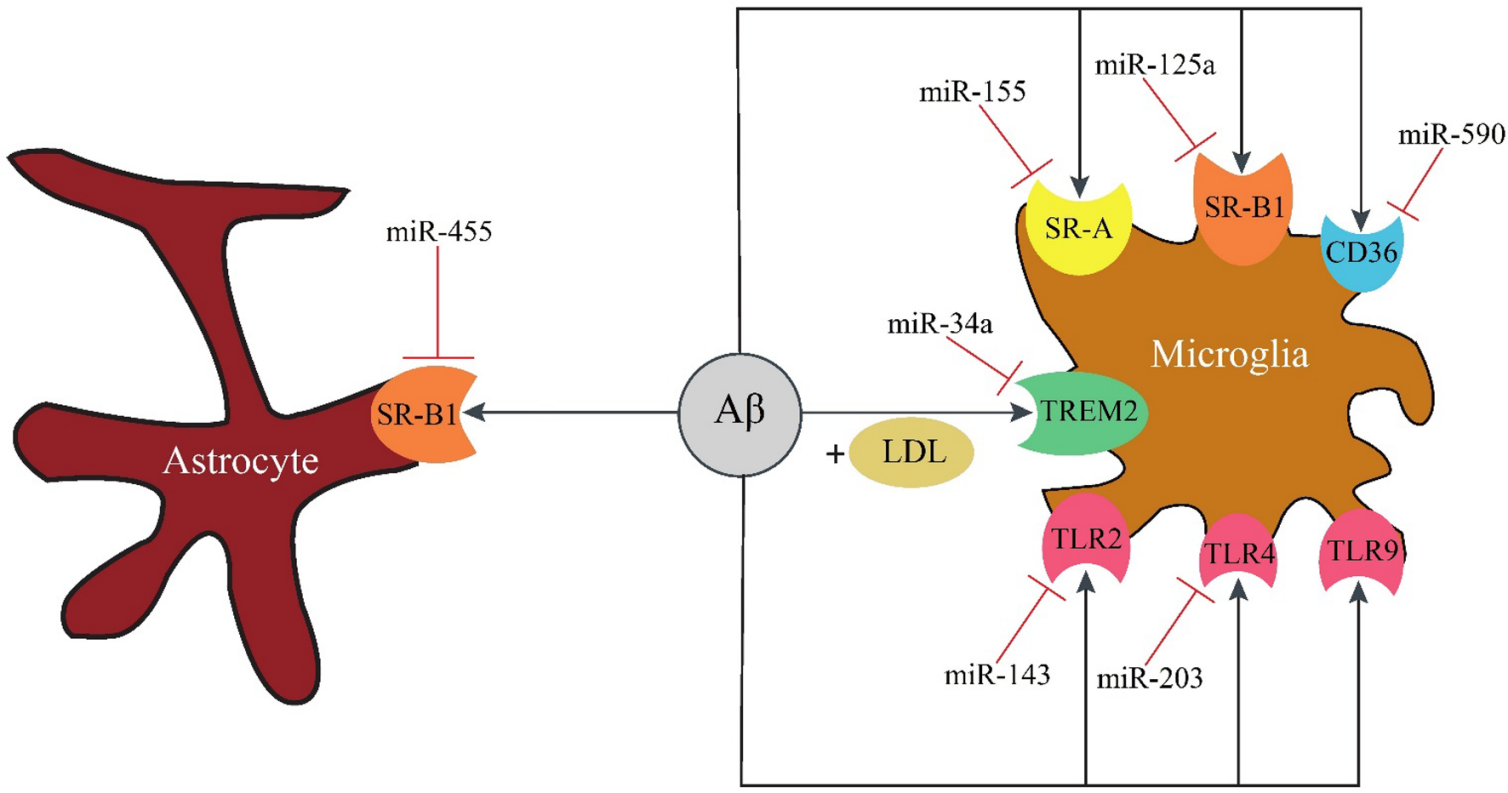
Receptor-mediated Aβ phagocytosis. The immune microglial cells and astrocytes reduce the load of Aβ in the brain by phagocytosis mediated by surface receptors. Aβ is directly phagocytosed by toll-like receptors and scavenger receptors, while the presence of LDL is crucial for TREM2-dependent phagocytosis. *TLR2* toll like receptor 2, *TLR4* toll like receptor 4, *TLR9* toll like receptor 9, *TREM2* triggering receptor expressed on myeloid cells 2, *LDL* low density lipoprotein, *SR-A* scavenger receptor class A, *SR-B1* scavenger receptor class B type 1

Phagocytosis is an evolutionarily conserved process, critical for innate immunity. It has been shown that impaired immune response in AD negatively affects Aβ elimination [239]. Similarly, macrophage-dependent phagocytosis of Aβ is impaired in AD [240]. In this section we introduce receptors that are expressed on the surface of phagocytic cells, and involved in Aβ phagocytosis. These surface receptors are regulated by several miRNAs, some of which are shown in Fig. 5 and Table 5.

**Table 5:** MiRNAs and their downregulated mRNA targets in the receptor-mediated Aβ phagocytosis

MiRNA	Family	Gene	References
miR-203, miR-27a	Toll-like receptors	TLR4	[268, 269]
miR-143, miR-19, miR-146a		TLR2	[270–272]
miR-155	Scavenger receptors	SR-A	[273]
miR-185, miR-96, miR-223		SR-BI	[274]
miR-590		CD36	[275]

### Toll-like receptors

Toll-like receptors (TLRs) are a family of pattern recognition receptors (PRRs), and involved in innate immune recognition. There are at least ten TLRs in mammals, and though they have a high degree of structural similarity, their functions are distinct. TLRs are involved in the clearance of diffuse and fibrillar forms of Aβ through microglial activation [241]. Song et al. [242] showed that TLR2 deletion increased Aβ levels in the brain of APP transgenic mice which was accompanied with memory deficits. Consistent with these results, TLR4 mutation caused Aβ deposition and cognitive deficits in a mouse model of AD [243]. Frank et al. [244] detected increased mRNA levels of TLR2, TLR4, and TLR9 in a transgenic mouse model. Zhang et al. [245] found that miR-181c suppressed the activity of the luciferase reporter plasmid containing TLR4 3′-UTR by reducing TLR4 mRNA and protein expression in microglial cells. Consequently, miR-181c inhibited the downstream production of proinflammatory mediators. Table 5 listed the miRNAs that inhibit the expression of TLR2 and TLR4.

### Triggering receptor expressed on myeloid cells 2

Triggering receptor expressed on myeloid cells 2 (TREM2) is expressed on microglial cells and belongs to the immunoglobulin superfamily. This surface receptor has several ligands, including low density lipoproteins (LDL), ApoJ and ApoE. Yeh et al. [246] showed that microglial cells are capable of uptaking LDL-Aβ complexes in a TREM2-dependent manner. In a mouse model of AD, TREM2 enhanced Aβ42 phagocytosis in the primary microglia [247]. Thus Aβ levels were higher in TREM2-deficient mice [248]. Kober et al. [249] found that the ligand affinity of LDL-Aβ complex was reduced in the R47H and R62H variants of TREM2, leading to phagocytosis impairment and Aβ accumulation [246]. Jay et al. [250] detected that TREM2 was up-regulated on microglial cells that were clustered around Aβ deposits in a mouse model of AD and human AD tissues. Alexandrov et al. [251] showed that miR-34a down-regulated TREM2 expression leading to Aβ accumulation by impairing phagocytosis.

### Scavenger receptors

Scavenger receptors (SRs) are cell surface receptors that participate in the uptake of various polyanionic ligands. Based on their protein sequence, SRs are classified into 10 families (A-J). It has been shown that scavenger receptor class A (SR-A) and class B type 1 (SR-B1), as well as CD36 participate in Aβ clearance [252–254]. SR-A which is expressed on microglial cells and macrophages is implicated in Aβ phagocytosis [255]. Therefore SR-A deficiency reduced phagocytic activity of microglia and macrophages [256, 257], accelerated Aβ accumulation and consequently led to increased mortality in a mouse model of AD [258]. SR-B1 is expressed on microglial cells and astrocytes, mediates the binding of Aβ to microglia [259] and is implicated in the astrocyte-mediated clearance of Aβ [260]. In vivo studies indicated that SR-B1 deficiency promoted Aβ deposition [261]. CD36 which is found in a variety of cell types mediates macrophage and microglial response to Aβ [262]. In vitro studies demonstrated that CD36 deficiency decreased Aβ phagocytosis [263], while PPARγ-induced CD36 up-regulation enhanced Aβ phagocytosis in microglia [264]. Kouadir et al. [265] reported the increases in SR-B1 and CD36 expression by Aβ42, while Giunta et al. [266] reported the downregulation of CD36 in AD patients. Li et al. [267] showed that miR-758-5p significantly reduced mRNA and protein levels of CD36, and therefore attenuated cellular uptake of cholesterol.

## Heat shock proteins

Heat shock proteins (HSPs), a group of molecular chaperones repress molecular denaturation under stressful conditions. HSPs also prevent protein aggregation by binding to newly synthesized or misfolded proteins, thereby helping maintain protein homeostasis. According to their size and function, HSPs can be divided into two different families: classic HSPs with a molecular weight of 60 kD or more that possess an ATP-binding site, e.g., HSP90 and HSP70, and small HSPs with a molecular weight of 40 kD or less that are ATP-independent, e.g., HSP27. Initial studies showed that HSPs regulated microglial interactions with Aβ, substantiating the role of HSP90 and HSP70 in phagocytosis-dependent Aβ clearance [276]. Subsequent in vivo studies showed similar data, and demonstrated that microglial clearance of Aβ was facilitated by HSP90 in a rat brain [277], and that HSP70 over-expression decreased Aβ levels in a mouse model of AD [278]. Furthermore, Evans et al. [279] demonstrated that HSP90 and HSP70 could induce structural changes in Aβ oligomers that suppressed self-assembly. Similarly, Rivera et al. [280] found that HSP70 prevented Aβ oligomerization and consequently reduced Aβ-induced toxicity in cultured neurons. HSP27 was also able to bind Aβ40, reducing its formation into mature fibrils [281]. Therefore, HSP27 protects neurons against Aβ [282]. On the other hand, Aβ could enhance the expression of HSP27 and HSP70 in neuronal cultures [283, 284]. Table 6 specified the miRNAs that inhibit HSPs expression.

**Table 6:** MiRNAs and their downregulated HSP targets

MiRNA	Family	Gene	References
miR-1, miR-134	Heat shock proteins	HSP90	[285, 286]
miR-142-3p, miR-34a		HSP70	[287, 288]
miR-214		HSP27	[289]

## Conclusion

Emerging evidences indicate that impaired Aβ clearance plays a crucial role in both EOAD and LOAD. Thus, understanding how Aβ is cleared from the brain might be of clinical relevance. Aβ removal from the brain occurs via various pathways: UPS, autophagy, proteolytic enzymes, transportation across the BBB and cellular uptake. Any disturbance of these pathways may lead to Aβ accumulation, resulting in the pathological process driving AD. Our present review shows that numerous miRNAs inhibit the translation of key molecules in these pathways, promoting the Aβ accumulation. This ability of miRNAs to target multiple mRNAs in the network of Aβ clearance make them to valuable therapeutic target molecules in AD. In particular, those miRNAs should be selected as target molecules that are involved in several pathways. As shown above, miR-34a and miR-29b may be attractive candidates for AD treatment because they inhibit at least three pathways leading to Aβ clearance. In the adult mammalian brain, miR-34a is highly expressed, and has been implicated in a range of neurodevelopmental and neuropathological processes. MiR-34a was reported to regulate neural stem/progenitor cell differentiation. High levels of this miRNA have been detected during epileptic seizures and ischemic stroke contributing to neuronal injury and death [290]. MiR-29b has been identified as a putative regulator of immunity. Moreover, ectopic expression of miR-29b promoted neuronal cell death, whereas its repression decreased cell death [291]. In summary, the research field on miRNAs is promising for therapeutic applications, not only for the treatment of AD but also for regenerative medicine. However, several obstacles prevent their utility in the clinic, of which the accurate determination of their expression levels might be a critical point [292]. Indeed, due to the lack of consensus on the reference controls, the appropriate normalization approach should be validated in each experimental study [293, 294].

## Data Availability

Not applicable.
